# ﻿Six new species of *Cryptochironomus* Kieffer (Diptera, Chironomidae) from the Nearctic region

**DOI:** 10.3897/zookeys.1200.119225

**Published:** 2024-05-09

**Authors:** Wen-Bin Liu, Cheng-Yan Wang, Ya-Ning Tang, Ying Wang, Wen-Xuan Pei, Chun-Cai Yan

**Affiliations:** 1 Tianjin Key Laboratory of Conservation and Utilization of Animal Diversity, Tianjin Normal University, Tianjin, 300387, China Tianjin Normal University Tianjin China

**Keywords:** Adult male, diagnostic characters, hypopygium, key, Nematocera, taxonomy, US

## Abstract

Six new species of *Cryptochironomus* Kieffer, 1918, *C.absum* Liu, **sp. nov.**, *C.beardi* Liu, **sp. nov.**, *C.dentatus* Liu, **sp. nov.**, *C.ferringtoni* Liu, **sp. nov.**, *C.parallelus* Liu, **sp. nov.** and *C.taylorensis* Liu, **sp. nov.**, are described and illustrated based on adult males. The specimens were collected from various water systems in the United States and preserved by Dr. Leonard Charles Ferrington Jr. An updated key to adult males of all known *Cryptochironomus* species in the Nearctic region is also provided.

## ﻿Introduction

The genus *Cryptochironomus* was erected by Kieffer in 1918, with Chironomus (Cryptochironomus) chlorolobus Kieffer, 1918 as type species. The adult males of this genus are distinguished by having a finger-shaped inferior volsella which lacks microtrichia, and is often completely covered by the small superior volsella (Cranston et al. 1989). The larvae primarily inhabit still waters, ranging from moderately eutrophic to super eutrophic conditions, making them a resilient species in environmental monitoring (Curry 1958). The genus comprises over 140 valid species and has a global distribution. All life stages of the genus have been studied by numerous authors (Townes 1945; Roback 1957; Curry 1958; Shilova 1966; Beck and Beck 1969; Sæther 1977, 2009; Sasa and Kikuchi 1995; Sasa 1998; Zorina 2000; Makarchenko et al. 2005; Silva et al. 2010; Yan et al. 2016, 2018).

The systematic review of *Cryptochironomus* and a key to all known males in the Nearctic region were supported by Townes (1945). Sæther (2009) compiled keys for all stages of the genus in the Nearctic region, which were subsequently updated by Silva et al. (2010). Currently, 14 species of the genus are known in the Nearctic region: *C.argus* Roback, *C.blarina* Townes, *C.conus* Mason, *C.curryi* Mason, *C.digitatus* (Malloch), *C.eminentia* Mason, *C.fulvus* (Johannsen), *C.imitans* Sæther, *C.parafulvus* Beck & Beck, *C.ponderosus* (Sublette), *C.ramus* Mason, *C.scimitarus* Townes, *C.sorex* Townes and *C.stylifera* (Johannsen). In the present study, six new species are described and illustrated based on adult males. An updated key to adult males of the genus in the Nearctic region is also provided.

## ﻿Materials and methods

The morphology and terminology are based on Sæther (1980). The material examined was mounted on slides using the procedure outlined by Sæther (1969). When three or more specimens were measured, the measurements are provided as the range and mean, with the number of observed specimens in parentheses if it differs from the number (n) stated at the beginning of the description. The specimens examined in this study are preserved by Dr. Leonard Charles Ferrington Jr. and deposited in the University of Minnesota Insect Collection (**UMSP**), St. Paul, Minnesota, U.S.A. All type specimens are stored in UMSP.

## ﻿Taxonomy

### ﻿ Cryptochironomus absum

Taxon classificationAnimaliaDipteraChironomidae

Liu sp. nov.

3C416028-2792-5FBB-8C35-CCE2FC0FC600

https://zoobank.org/1E37808F-5EC2-483E-A84E-61D250652987

Figs 1 , 2 , 3

#### Type material.

***Holotype*.** one male, USA, Chamberlain South, Dakota State, Lake Francis Case Elm Creek, 43°56'57"N, 99°31'40"W, 3.IX.1971, light trap, leg: Patrick l. Huson.

#### Diagnostic characters.

AR 2.58; frontal tubercles absent; posterior margin of tergite IX arc-shaped; anal point slightly constricted at base, wider at apex; superior volsella oval-shaped, covered with microtrichia at 1/3 distance from apex; inferior volsella completely covered by superior volsella; gonostylus straight, parallel-sided, tapering to the apex.

#### Description.

**Male** (*n* = 1).

Total length 4.47 mm. Wing length 2.02 mm. Total length/wing length 2.22. Wing length/length of profemur 2.16.

***Coloration*.** Thorax yellowish brown. Femora of front legs yellowish brown, tibiae dark brown, tarsi lost; femora and tibiae of mid and hind legs light yellowish brown; mid and hind legs with tarsi I yellowish brown except for dark yellowish brown at both ends, tarsi II–V dark yellowish brown. Abdomen yellowish brown, hypopygium dark brown.

**Figure 1.**
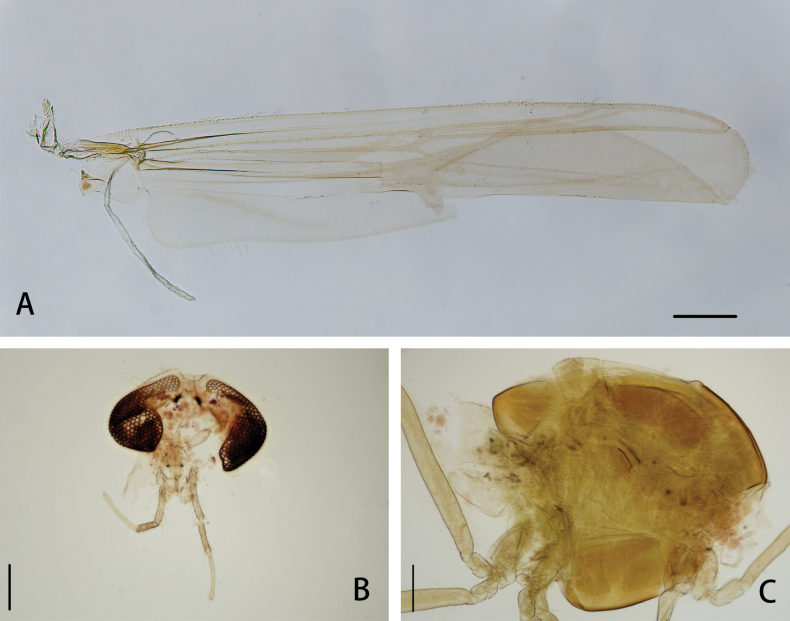
*Cryptochironomusabsum* Liu, sp. nov., holotype male **A** wing **B** head **C** thorax. Scale bars: 200 μm.

***Head*** (Fig. 1B). Antenna with 11 flagellomeres, ultimate flagellomere 851 μm long. AR 2.58. Frontal tubercles absent. Temporal setae 20. Clypeus with 18 setae. Tentorium 177 μm long, 47 μm wide. Palpomere lengths (in μm): 36; 60; 179; 151; 203; Pm5/Pm3 1.13.

***Thorax*** (Fig. 1C). Antepronotals bare; acrostichals 8; dorsocentrals 9; prealars 5. Scutellum with 16 setae.

***Wing*** (Figs 1A, 3C). VR 1.08. R with 22 setae, R_1_ with 11 setae, R_4+5_ with 17 setae. Brachiolum with three setae. Squama with 14 fringed setae.

***Legs*.** Front tibia with three subapical setae, 143 μm, the remaining lost. Mid legs with two tibial spurs, 42 μm long, the other lost, tibial combs 34 μm and 56 μm wide. Hind legs with two tibial spurs, 23 μm and 42 μm long, tibial combs 44 μm and 87 μm wide. Tarsus I of mid leg with three sensilla chaetica; tarsus I of hind leg with three sensilla chaetica. Lengths (in μm) and proportions of legs as in Table 1.

**Table 1.** Lengths (in μm) and proportions of legs of *Cryptochironomusabsum* Liu, sp. nov., adult male (*n* = 1).

	**fe**	**ti**	**ta_1_**	**ta_2_**	**ta_3_**
P_1_	934	721	-	-	-
P_2_	876	738	516	230	155
P_3_	964	973	730	367	301
	**ta_4_**	**ta_5_**	**LR**	**BV**	**SV**
P_1_	-	-	-	-	-
P_2_	102	89	0.70	3.70	3.13
P_3_	168	110	0.75	2.82	2.65

**Figure 2.**
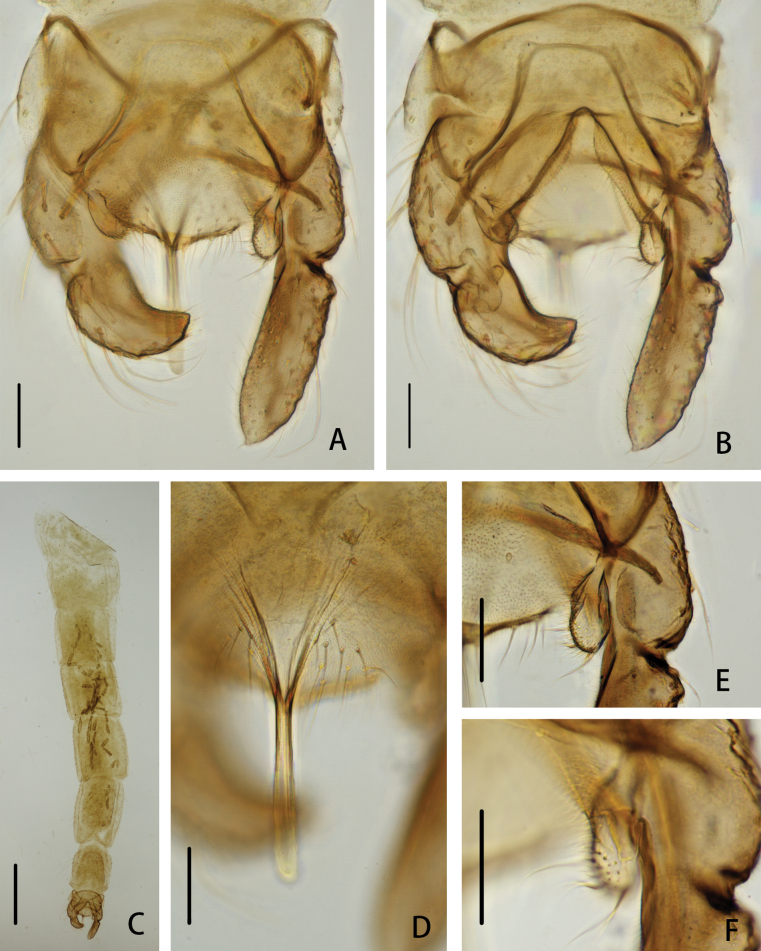
*Cryptochironomusabsum* Liu, sp. nov., holotype male **A** hypopygium, dorsal view **B** hypopygium, ventral view **C** abdomen **D** anal point **E** superior volsella **F** inferior volsella. Scale bars: 50 μm (**A, B, D–F**); 500 μm (**C**).

**Figure 3.**
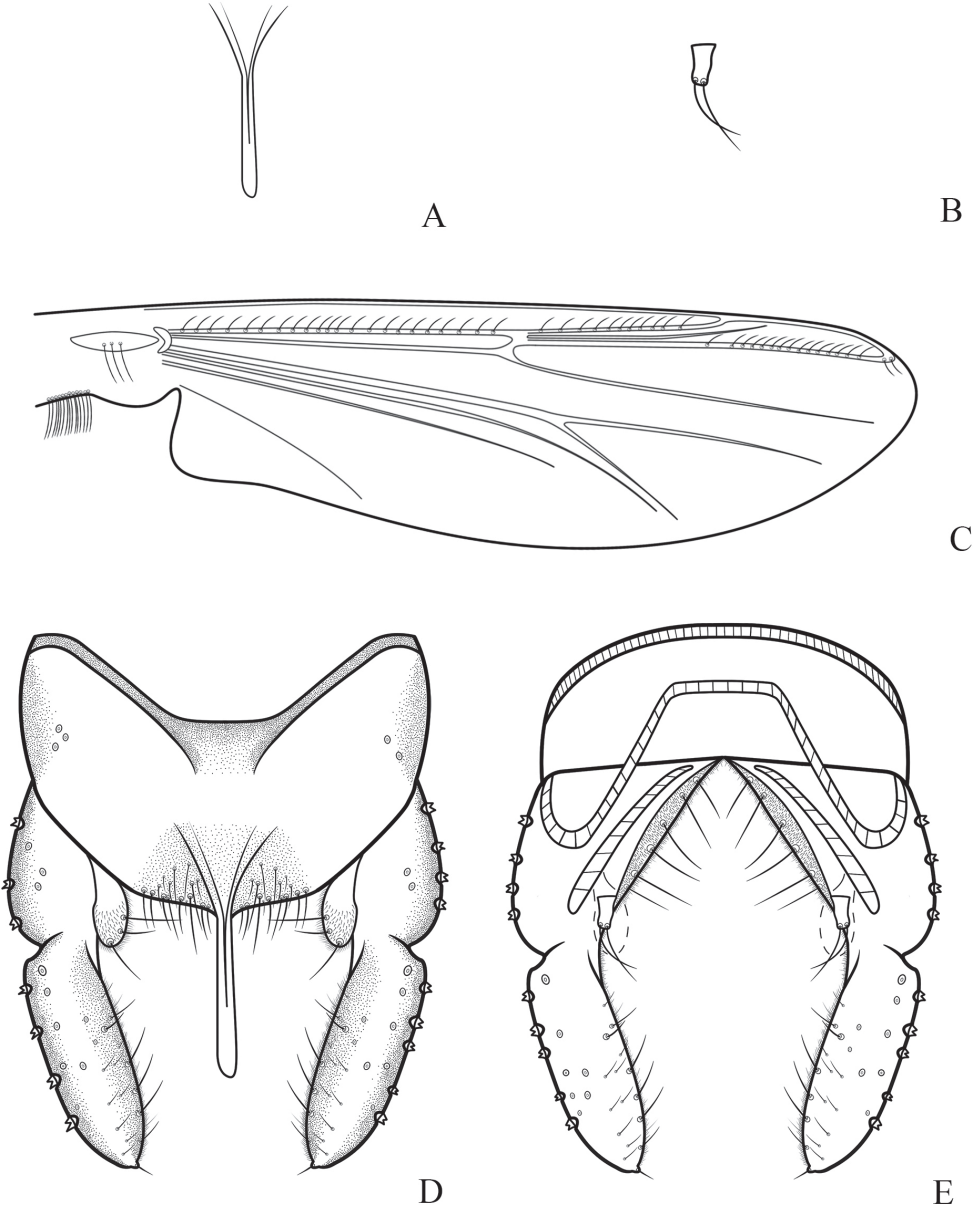
*Cryptochironomusabsum* Liu, sp. nov., holotype male **A** anal point **B** inferior volsella **C** wing **D** hypopygium, dorsal view **E** hypopygium, ventral view.

***Hypopygium*** (Figs 2, 3A, B, D, E). The posterior margin of tergite IX is arc-shaped and bears 32 setae, located dorsally and ventrally near the base of the anal point. Laterosternite IX has three setae. The anal point is 107 μm long, slightly constricted at the base, wider at the apex, and lacks lateral setae and microtrichia. The anal tergite bands are V-shaped and fused in the middle. The phallapodeme measures 134 μm long, and the transverse sternapodeme is 69 μm long. The superior volsella is oval-shaped, 56 μm long and 24 μm wide, covered with microtrichia at 1/3 distance from the apex, and has three long setae apically. The inferior volsella is finger-shaped, 22 μm long, bears two setae at the apex, is completely covered by the superior volsella, and lacks microtrichia. The gonocoxite measures 166 μm long and bears six strong setae along the inner margin. The gonostylus is 157 μm long, straight, parallel-sided, tapers to the apex, bears five setae along the inner margin, and has one single seta at the apex. HR 1.06. HV 2.85.

#### Etymology.

From the Latin, *absum*, absent, referring to the character of frontal tubercles absent, adjective in the nominative singular.

#### Remarks.

*Cryptochironomusabsum* Liu, sp. nov. is similar to *C.conus* Mason, 1985 in having anal tergite bands, anal point, and superior volsella with similar shapes. However, it can be distinguished from *C.conus* by the following combination of characters: wing length of 2.02 mm, absence of frontal tubercles, inferior volsella with a finger-shaped appearance, and gonostylus straight in this new species. In contrast, *C.conus* has a wing length of 5.1–5.3 mm, distinct frontal tubercles, an inferior volsella with a tuberculate appearance and a protrusion at the base, and a curved gonostylus.

### ﻿ Cryptochironomus beardi

Taxon classificationAnimaliaDipteraChironomidae

Liu sp. nov.

D278DE0F-3C13-5C27-9E2C-9BC211ED3766

https://zoobank.org/916D7812-69CB-4318-BAAF-3438C95DC17D

Figs 4 , 5 , 6

#### Type material.

***Holotype*.** one male, USA, New Mexico State, Rio Grande Otowi Bridge between Santa Fe and Los Alamos, 35°87'48"N, 99°31'40"W, 16.VII.1976, sweep net, leg: Melvin. Beard. ***Paratype*.** one male, North America, San Juan River at Farmington, 18.VII.1976, malaise trap, M. Beard.

#### Diagnostic characters.

Thorax pale yellow, with yellow-brown spots; anal point widest at base, constricted slightly at 1/3 distance from base, apically rounded; anal tergite bands V-shaped, fused in the middle; superior volsella oval-shaped, stretching upward at base, swelling at apex; inferior volsella columnar, ~ 2× as long as wide, with slender extension at base, bearing two long setae at apex, free microtrichia; gonostylus protruded at base, slightly curved in the middle, tapered to the apex.

#### Description.

**Male** (*n* = 2, unless stated).

Total length 5.03–5.10, 5.07 mm. Wing length 2.43–2.65, 2.54 mm. Total length/wing length 1.89–2.10, 2.00. Wing length/length of profemur 2.13 (1).

***Coloration*.** Thorax pale yellow, with yellowish brown spots. Femora of front legs yellowish brown except for dark yellow-brown at ends, tibiae and tarsomeres dark brown; femora of mid and hind legs yellowish brown, tarsi I yellowish brown with dark brown in distal; tarsi II–V dark yellowish brown. Abdomen pale yellow, hypopygium yellowish brown.

**Figure 4.**
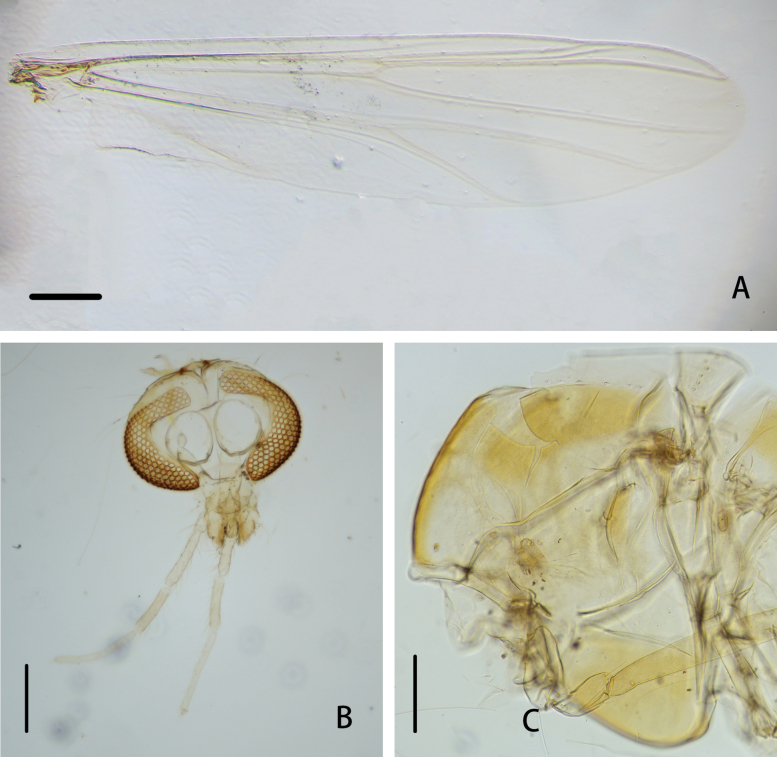
*Cryptochironomusbeardi* Liu, sp. nov., holotype male **A** wing **B** head **C** thorax. Scale bars: 200 μm.

***Head*** (Figs 4B, 6A). Antenna with 11 flagellomeres, ultimate flagellomere 972 (1) μm long. AR 2.63 (1). Frontal tubercles conical, 18 μm high, 5 μm width at base. Temporal setae 17–18, 18. Clypeus with 12–18, 15 setae. Tentorium 167–173, 170 μm long, 45–50, 48 μm wide. Palpomere lengths of one specimen (in μm): 40; 61; 198; 171; 275; Pm5/Pm3 1.39.

***Thorax*** (Fig. 4C). Antepronotals with three setae, acrostichals 6–9, 8; dorsocentrals 8–10, 9; prealars 6. Scutellum with 14 setae.

***Wing*** (Figs 4A, 6D). VR 1.09. R with 26–28, 27 setae, R_1_ with 19–22, 21 setae, R_4+5_ with 22–25, 24 setae. Brachiolum with two setae. Squama with 8–9, 9 setae.

***Legs*.** Front tibia with three subapical setae, 144 (1) μm, 151 (1) μm and 162 (1) μm. Combs of mid tibia 38–42, 40 μm wide with 22–27, 25 μm long spur, and 52–58, 55 μm wide with 30–40, 35 μm long spur; combs of hind tibia 47–48, 48 μm wide with 20–25, 23 μm long spur, 81–88, 85 μm wide with 38–42, 40 μm long spur. Tarsus I of mid leg with three sensilla chaetica. Tarsus I of hind leg with four sensilla chaetica. Lengths (in μm) and proportions of legs as in Table 2.

**Table 2.** Lengths (in μm) and proportions of legs of *Cryptochironomusbeardi* Liu, sp. nov. adult males (*n* = 2).

	**fe**	**ti**	**ta_1_**	**ta_2_**	**ta_3_**
P_1_	1139 (1)	857 (1)	1442 (1)	648 (1)	528 (1)
P_2_	998–1121, 1060	913–950, 932	579 (1)	266 (1)	204 (1)
P_3_	1156–1226, 1191	1185–1305, 1245	832 (1)	397 (1)	299 (1)
	**ta_4_**	**ta_5_**	**LR**	**BV**	**SV**
P_1_	498 (1)	170 (1)	1.68 (1)	1.86 (1)	1.38 (1)
P_2_	121 (1)	107 (1)	0.63 (1)	3.57 (1)	3.30 (1)
P_3_	185 (1)	112 (1)	0.70 (1)	3.20 (1)	2.81 (1)

**Figure 5.**
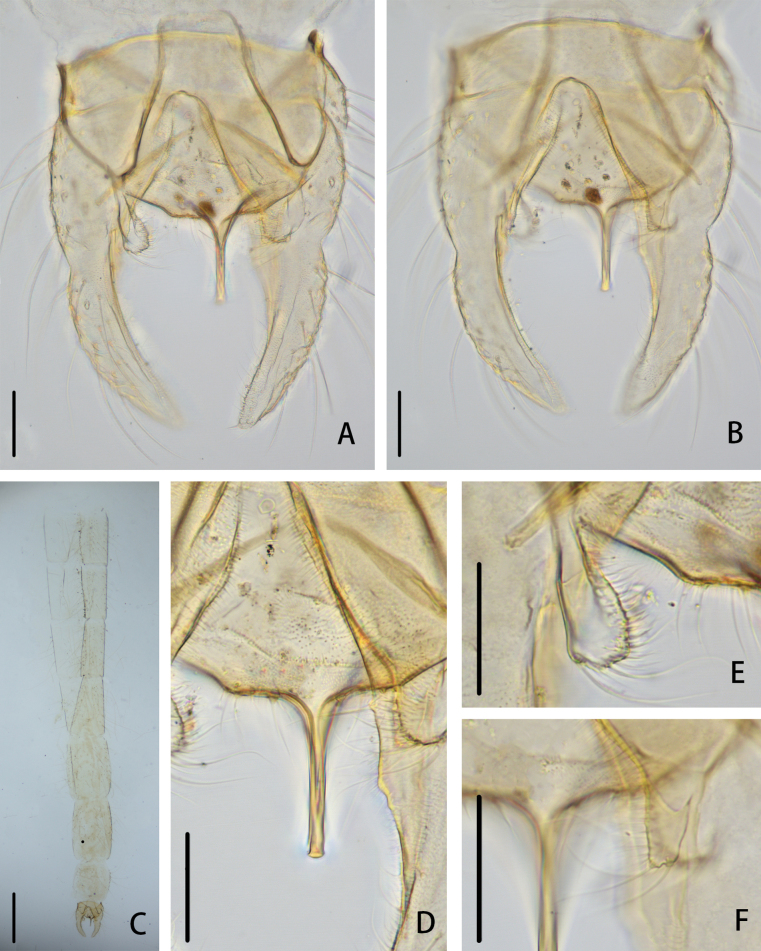
*Cryptochironomusbeardi* Liu, sp. nov., holotype male **A** hypopygium, dorsal view **B** hypopygium, ventral view **C** abdomen **D** anal point **E** superior volsella **F** inferior volsella. Scale bars: 50 μm (**A, B, D–F**); 500 μm (**C**).

**Figure 6.**
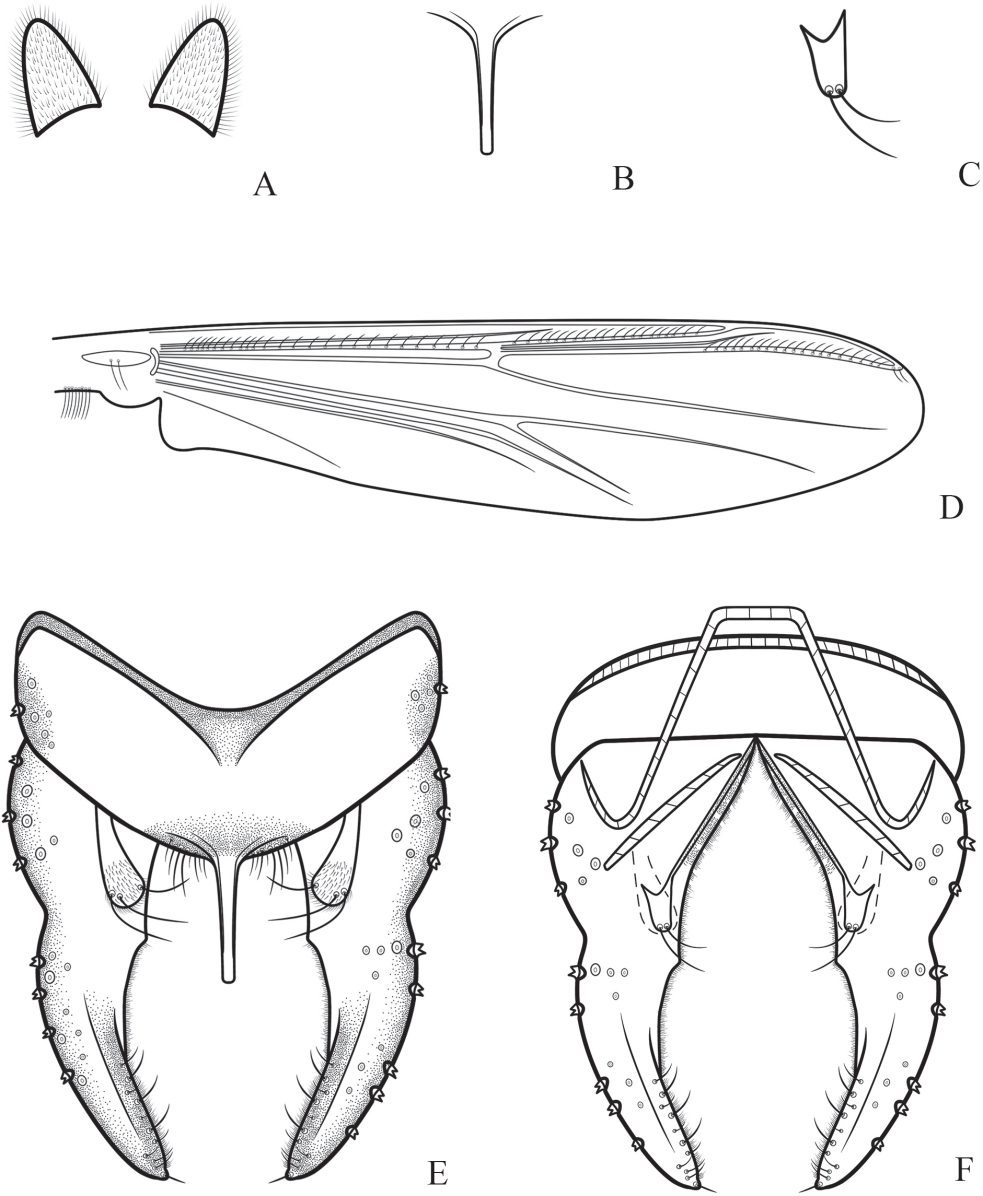
*Cryptochironomusbeardi* Liu, sp. nov., holotype male **A** frontal tubercles **B** anal point **C** inferior volsella **D** wing **E** hypopygium, dorsal view **F** hypopygium, ventral view.

***Hypopygium*** (Figs 5, 6B, C, E, F). The posterior margin of tergite IX is shoulder-shaped and bears 16–18, 17 setae located dorsally and ventrally near the base of the anal point. Laterosternite IX has 5–6, 6 lateral setae. The anal point is 70–80, 75 μm long, widest at the base, slightly constricted at 1/3 distance from the base, and apically rounded. The anal tergite bands are V-shaped and fused in the middle. The phallapodeme measures 113–115, 114 μm long, and the transverse sternapodeme is 57–61, 59 μm long. The superior volsella is oval-shaped, 45–50, 48 μm long, 22–29, 26 μm wide, stretching upward at the base, swelling at the apex, covered with microtrichia, and bears three strong setae at the apex. The inferior volsella is columnar, 25–31, 28 μm long, ~ 2× as long as wide, with a slender extension at the base, bearing two long setae at the apex, and lacking free microtrichia. The gonocoxite measures 151–164, 158 μm long and bears six strong setae along the inner margin. The gonostylus is 162–166, 164 μm long, widest and protruded at the base, slightly curved in the middle, tapered towards the apex, bears five short setae along the inner margin and one stronger seta at the apex.

#### Etymology.

Name after M. Beard, for the collector of the materials; noun in nominative case.

#### Remarks.

*Cryptochironomusbeardi* Liu, sp. nov. resembles *C.maculus* Yan & Wang, 2016 in having gonostylus and inferior volsella with similar shapes. However, it can be distinguished from *C.maculus* by the following combination of characters: mid and hind legs with tarsi I yellowish brown base, dark brown distally, anal point apically rounded, superior volsella stretching upward, gonocoxite concave with gonostylus obviously in the former; whereas in *C.maculus*, the mid and hind legs have the tarsi I yellowish green with dark brown mark on basal portion, anal point apically pointed, superior volsella not stretching, gonocoxite fused with gonostylus completely.

### ﻿ Cryptochironomus dentatus

Taxon classificationAnimaliaDipteraChironomidae

Liu sp. nov.

A32EAD98-AC31-5AA8-AA63-AD6FD1994E45

https://zoobank.org/A506086F-D86F-4EC6-ADBC-5B1FDFBD9B43

Figs 7 , 8 , 9

#### Type material.

***Holotype*.** One male, USA, New Mexico State, Guadalupe County, Pecos River, Puerto de Luna below Diversion Dam, 33°04'06"N, 104°26'79"W, 20.VIII.1991, light trap, leg: Lensky & Doles. ***Paratypes*.** 8 males, USA, Guadalupe Country, Pecos River, Puerto de Luna below Diversion Dam, 20.VIII.1991, light trap, leg: Lensky & Doles.

#### Diagnostic characters.

Frontal tubercles conical; tergite IX saddle-shaped at the posterior margin; anal point parallel-sided with rounded apex; superior volsella crescent-like; inferior volsella finger-shaped, dentate at base; gonostylus curved slightly at 1/3 distance from base, swelling at distal 1/3.

#### Description.

**Male** (*n* = 9, unless stated).

Total length 4.58–5.30, 4.91 mm. Wing length 2.05–2.29, 2.16 mm. Total length/wing length 2.14–2.31, 2.26. Wing length/length of profemur 2.04–2.61, 2.23.

***Coloration*.** Thorax yellowish brown. Femora of front legs yellowish brown, tibiae and tarsi dark brown; femora and tibiae of mid and hind legs yellowish brown, tarsi I yellowish brown, tarsi II–V yellowish brown to dark brown gradually. Abdomen yellowish brown, hypopygium dark brown.

**Figure 7.**
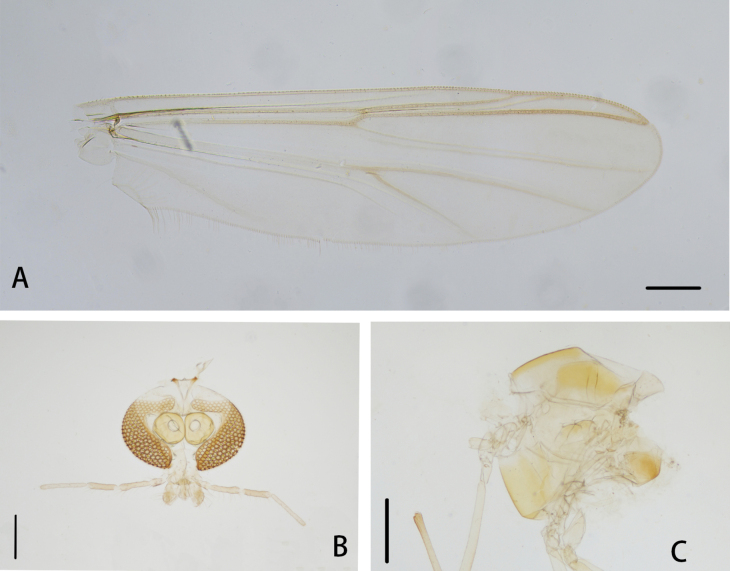
*Cryptochironomusdentatus* Liu, sp. nov., holotype male **A** wing **B** head **C** thorax. Scale bars: 200 μm.

***Head*** (Figs 7B, 9A). Antenna with 11 flagellomeres, ultimate flagellomere 770–879, 829 (4) μm long. AR 2.48–2.76, 2.61 (4). Frontal tubercles conical, 20–31, 25 μm high, 12–16, 14 μm width at base. Temporal setae 15–21, 18. Clypeus with 10–16, 12 setae. Tentorium 115–170, 147 μm long, 30–46, 40 μm wide. Palpomere lengths (in μm): 33–47, 41; 52–76, 67; 136–174, 158; 140–157, 148; 201–232, 211; Pm5/Pm3 1.18–1.48, 1.35.

***Thorax*** (Fig. 7C). Antepronotals bare; acrostichals 5–8, 6; dorsocentrals 8–12, 10; prealars 4. Scutellum with 14–18, 16 setae.

Wing (Figs 7A, 9D). VR 1.08–1.12, 1.10. R with 14–18, 17 setae, R_1_ with 12–15, 13 setae, R_4+5_ with 19–25, 22 setae. Brachiolum with two setae. Squama with ten setae.

***Legs*.** Front tibia with three subapical setae, 87–111, 98 (6) μm, 110–123, 115 (6) μm, 117–133, 124 μm. Combs of mid tibia 37–47, 43 μm wide with 23–34, 27 μm long spur, and 34–54, 46 μm wide with 32–45, 39 μm long spur; combs of hind tibia 30–49, 40 μm wide with 27–40, 31 μm long spur, 80–90, 83 μm wide with 34–48, 42 μm long spur. Tarsus I of mid leg with seven sensilla chaetica; tarsus I of hind leg with five sensilla chaetica. Lengths (in μm) and proportions of legs as in Table 3.

**Table 3.** Lengths (in μm) and proportions of legs of *Cryptochironomusdentatus* Liu, sp. nov., adult males (*n* = 9, unless stated).

	**fe**	**ti**	**ta_1_**	**ta_2_**	**ta_3_**
P_1_	877–1002, 963	739–797, 770	1223–1361, 1270 (4)	596–638, 621 (4)	484–524, 505 (4)
P_2_	831–978, 925 (6)	788–872, 826	461–520, 490	234–264, 248	175–205, 190
P_3_	987–1080, 1038 (6)	1071–1182, 1116	712–795, 748 (6)	370–413, 392 (6)	306–343, 318 (6)
	**ta_4_**	**ta_5_**	**LR**	**BV**	**SV**
P_1_	362–412, 394 (4)	174–191, 185 (4)	1.61–1.71, 1.65 (4)	1.72–1.81, 1.76 (4)	1.23–1.46, 1.37 (4)
P_2_	115–134, 124	100–111, 106	0.56–0.62, 0.59	3.06–3.53, 3.34 (6)	3.41–3.83, 3.59 (6)
P_3_	168–188, 179 (6)	122–126, 124 (6)	0.66–0.68, 0.67 (6)	2.82–2.88, 2.86 (6)	2.82–2.93, 2.88 (6)

**Figure 8.**
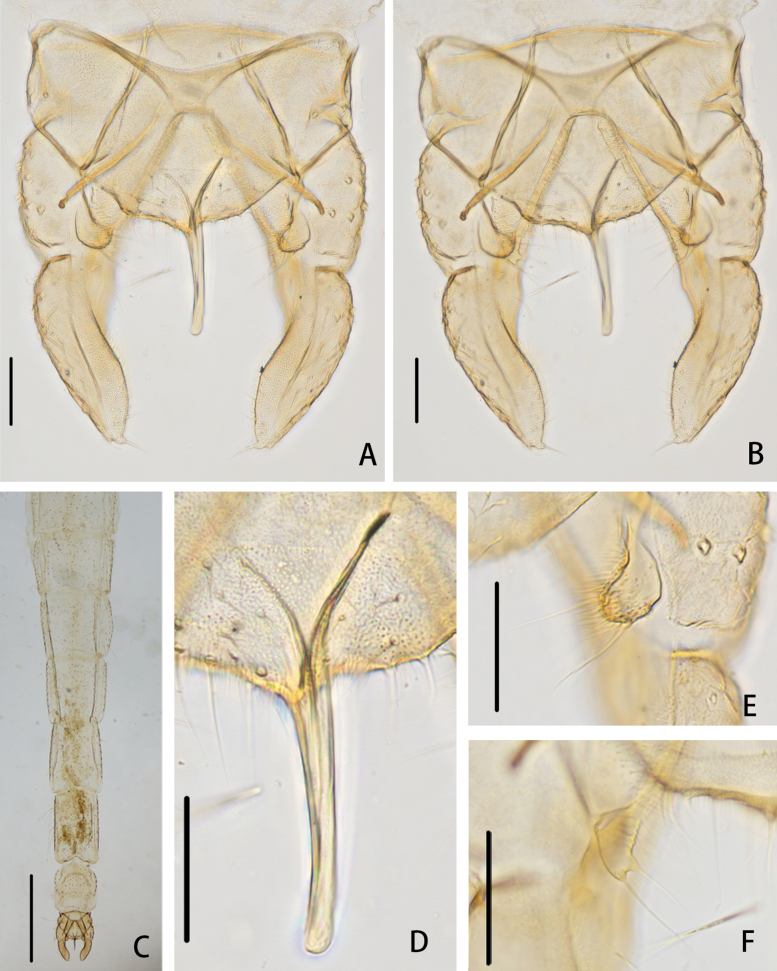
*Cryptochironomusdentatus* Liu, sp. nov., holotype male **A** hypopygium, dorsal view **B** hypopygium, ventral view **C** abdomen **D** anal point **E** superior volsella **F** inferior volsella. Scale bars: 50 μm (**A, B, D–F**); 500 μm (**C**).

**Figure 9.**
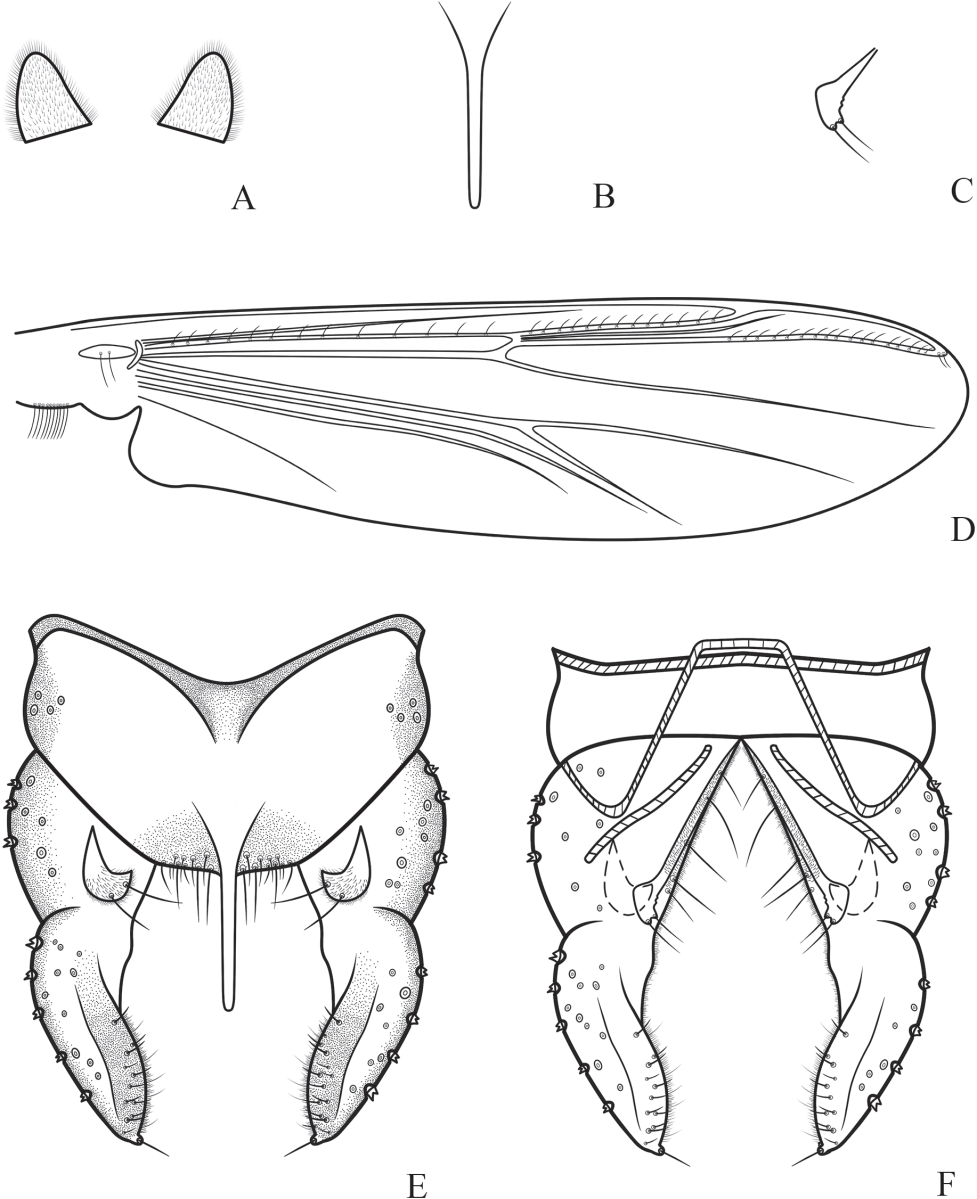
*Cryptochironomusdentatus* Liu, sp. nov., holotype male **A** frontal tubercles **B** anal point **C** inferior volsella **D** wing **E** hypopygium, dorsal view **F** hypopygium, ventral view.

***Hypopygium*** (Figs 8, 9B, C, E, F). The posterior margin of tergite IX is shoulder-shaped and bears 20–36, 30 setae. Laterosternite IX has 4–5, 4 setae. The anal point measures 77–87, 81 μm long and is parallel-sided with a rounded apex. The anal tergite bands are V-shaped and fused in the middle. The phallapodeme measures 115–135, 126 μm long, and the transverse sternapodeme is 64–90, 73 μm long. The superior volsella is crescent-like, 37–55, 48 μm long, 16–27, 21 μm wide, covered with microtrichia, and bears two strong setae at the apex. The inferior volsella is finger-shaped, 19–23, 21 μm long, dentate at the base, bearing two setae at the apex, and lacking free microtrichia. The gonocoxite measures 167–175, 170 μm long and bears five strong setae along the inner margin. The gonostylus is 150–157, 155 μm long, curved slightly at 1/3 distance from the base, swelling at the distal 1/3, with a small protrusion at the apex and bearing one apical seta. HR 1.08–1.12, 1.10. HV 2.96–3.38, 3.18.

#### Etymology.

From the Latin, *dentatus*, dentate, tooth-like, referring to the shape of the base of inferior volsella, adjective in the nominative singular.

#### Remarks.

*Cryptochironomusdentatus* Liu, sp. nov. bears resemblance to *C.fulvus* Johannsen, 1905 due to its similar frontal tubercles, anal point, and superior volsella. However, *C.dentatus* Liu, sp. nov. can be distinguished from *C.fulvus* by the following combination of characters: absence of spots on the thorax, tergite IX with a shoulder-shaped posterior margin, inferior volsella with a finger-shape and dentate base; whereas *C.fulvus*, has dark brown spots on the thorax, tergite IX with a conical posterior margin, and inferior volsella with a tuberculate and non-dentate base.

### ﻿ Cryptochironomus ferringtoni

Taxon classificationAnimaliaDipteraChironomidae

Liu sp. nov.

ECE142BA-22BE-5238-8557-228345404BC6

https://zoobank.org/C4891774-65E5-429C-9F82-452A5829F324

Figs 10 , 11 , 12

#### Type material.

***Holotype*.** One male, USA, South Dakota State, Springfield, Lewis and Clark Lake, Boat Basin, 42°87'33"N, 97°49'02"W, 13–17.VII.1964, leg: Pat Hadson. ***Paratypes*.** 3 males, Ohio, Cincinnati, Federal Water Quality Association, lab on the window, 26.X.1970, leg: W. T. Mason. 2 males, Springfield South Dakota, Boat Basin, Springfield, Lewist Clank Lake, 13–17.VII.1964, leg: Pat Hadson.

#### Diagnostic characters.

Tergite IX with slightly cone-like posterior margin; anal point narrow at basal 1/3, expanded at 1/3 of the apex, apically rounded; superior volsella crescent-like, with hook-like extension at the base, apically rounded; inferior volsella triangular widest at base, apex with a small protrusion; the junction of gonostylus and gonocoxite concaved obviously; gonostylus widest at basal 1/3, tapered to the apex.

#### Description.

**Male** (*n* = 6, unless stated).

Total length 3.29–3.91, 3.58 mm. Wing length 1.47–1.69, 1.61 mm. Total length/wing length 2.12–2.37, 2.23. Wing length/length of profemur 1.81–2.24, 2.02.

***Coloration*.** Thorax yellowish brown. Femora of front legs yellowish brown, tibiae and tarsi lost; mid and hind legs yellowish brown. Abdomen pale yellow, hypopygium yellowish brown.

**Figure 10.**
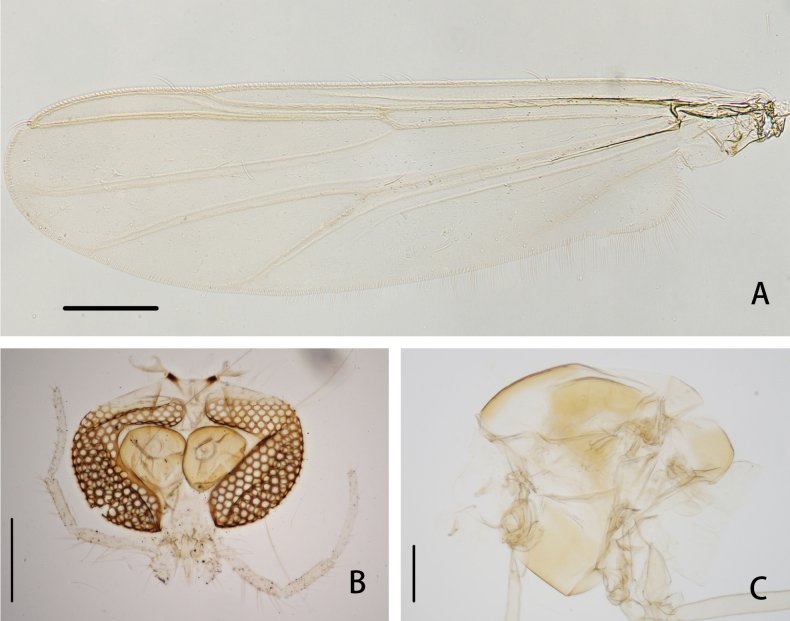
*Cryptochironomusferringtoni* Liu, sp. nov., holotype male **A** wing **B** head **C** thorax. Scale bars: 200 μm.

***Head*** (Fig. 10B). Antenna with 11 flagellomeres, ultimate flagellomere 698–754, 724 (4) μm long. AR 2.29–2.49, 2.41 (4). Frontal tubercles absent. Temporal setae 14–17, 15. Clypeus with 12–18, 14 setae. Tentorium 126–153, 140 μm long, 31–37, 34 μm wide. Palpomere lengths (in μm): 21–36, 32; 41–56, 49; 126–155, 144; 114–144, 132; 174–232, 204; Pm5/Pm3 1.18–1.55, 1.40.

***Thorax*** (Fig. 10C). Antepronotals bare; acrostichals 4–7, 5; dorsocentrals 4–8, 7; prealars 4–5, 5. Scutellum with 8–14, 11 setae.

***Wing*** (Figs 10A, 12C). VR 1.13–1.19, 1.16. R with 12–17, 15 setae, R_1_ with 10–16, 14 setae, R_4+5_ with 15–21, 19 setae. Brachiolum with 2–3, 2 setae. Squama bare.

***Legs*.** Front tibia with three subapical setae, 113–132, 123 (3) μm, 124–127, 126 (3) μm, 128–144, 135 (3) μm. Mid legs with two spurs, 16–23, 20 μm and 20–34, 27 μm long, tibial comb 20–39, 30 μm and 27–40, 32 μm wide. Spurs of hind tibia 19–30, 26 μm and 30–45, 36 μm long, tibial comb 30–43, 36 μm and 50–83, 67 μm wide. Tarsus I of mid leg with 4–5, 4 sensilla chaetica, tarsus I of metapedes leg with 3–4, 4 sensilla chaetica. Lengths (in μm) and proportions of legs as in Table 4.

**Table 4.** Lengths (in μm) and proportions of legs of *Cryptochironomusferringtoni* Liu, sp. nov., adult males (*n* = 6, unless stated).

	**fe**	**ti**	**ta_1_**	**ta_2_**	**ta_3_**
P_1_	724–938, 800	483–559, 531	960 (1)	-	-
P_2_	633–730, 691	554–662, 613	389–468, 430	159–186, 172	112–134, 125
P_3_	610–797, 711	710–850, 795	566–638, 603 (4)	245–296, 269 (4)	204–238, 220 (4)
	**ta_4_**	**ta_5_**	**LR**	**BV**	**SV**
P_1_	-	-	1.81	-	-
P_2_	73–89, 80	62–74, 67	0.69–0.71, 0.70	3.73–3.99, 3.91	2.93–3.08, 3.04
P_3_	122–142, 131 (4)	80–91, 85 (4)	0.73–0.80, 0.76 (4)	2.87–3.12, 2.99 (4)	2.33–2.59, 2.50 (4)

**Figure 11.**
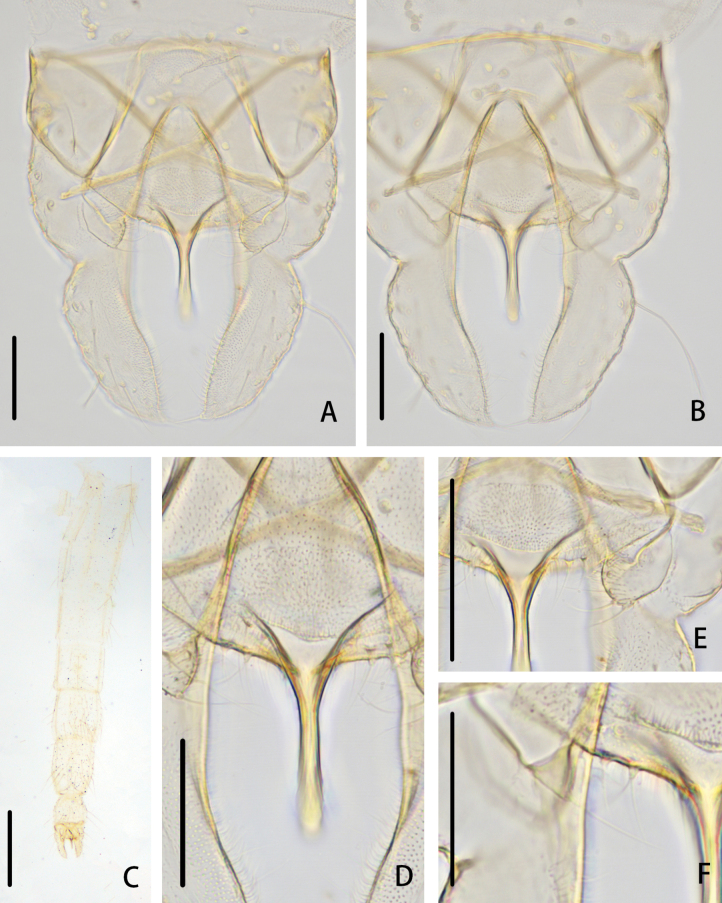
*Cryptochironomusferringtoni* Liu, sp. nov., holotype male **A** hypopygium, dorsal view **B** hypopygium, ventral view **C** abdomen **D** anal point **E** superior volsella **F** inferior volsella. Scale bars: 50 μm (**A, B, D–F**); 500 μm (**C**).

**Figure 12.**
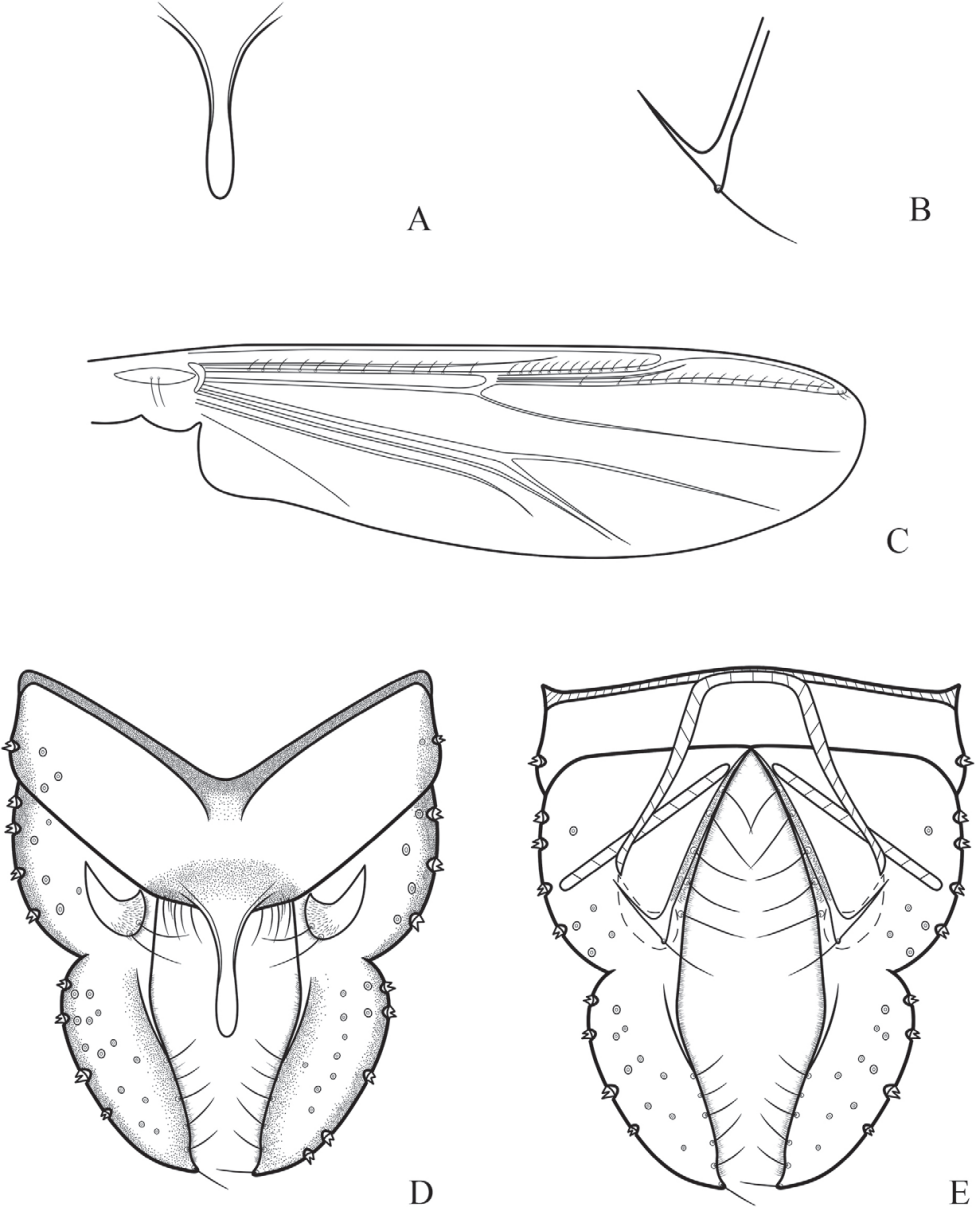
*Cryptochironomusferringtoni* Liu, sp. nov., holotype male **A** anal point **B** inferior volsella **C** wing **D** hypopygium, dorsal view **E** hypopygium, ventral view.

***Hypopygium*** (Figs 11, 12A, B, D, E). The posterior margin of tergite IX is slightly cone-like and bears 12–20, 16 setae located dorsally and ventrally near the base of the anal point. Laterosternite IX has 3–6, 4 lateral setae. The anal point measures 55–63, 59 μm long and is contracted at the basal 1/3, expanded at the 1/3 of the apex, and apically rounded. The anal tergite bands are V-shaped and jointed medially. The phallapodeme measures 93–102, 96 μm long, and the transverse sternapodeme is 43–67, 54 μm long. The superior volsella is crescent-like, 35–39, 37 μm long, 18–27, 22 μm wide, with a hook-like extension at the base, apically rounded, and bears two strong setae at the apex. The inferior volsella is triangular, 12–15, 13 μm long, widest at the base, tapered towards the apex, with a small protrusion at the apex and bearing one seta. The gonocoxite measures 105–124, 116 μm long. The junction of the gonostylus and gonocoxite is distinctively concave. The gonostylus is 104–126, 115 μm long, widest at the basal 1/3, tapered to the apex, and bears one stronger seta at the apex. HR 0.96–1.06, 1.01. HV 2.92–3.33, 3.13.

#### Etymology.

Name after Dr. Leonard Charles Ferrington Jr., for his outstanding contribution to the knowledge of Chironomidae taxonomy; noun in nominative case.

#### Remarks.

*Cryptochironomusferringtoni* Liu, sp. nov. bears resemblance to *C.rostratus* Kiffer, 1921 due to its similar shapes of the posterior margin of tergite IX and superior volsella. However, it can be distinguished from *C.rostratus* by the absence of frontal tubercles, contraction of the anal point at the basal 1/3 and expansion at the apical 1/3, and a triangular inferior volsella in *C.ferringtoni* Liu, sp. nov.; whereas *C.rostratus* frontal tubercles, a distally tapering or parallel-sided anal point, and a tuberculate inferior.

### ﻿ Cryptochironomus parallelus

Taxon classificationAnimaliaDipteraChironomidae

Liu sp. nov.

56306045-213F-53F2-BFF8-1B3283A26322

https://zoobank.org/C30E333F-00DF-49CB-B611-8BDD3E032EBB

Figs 13 , 14 , 15

#### Type material.

***Holotype*.** one male, USA, New Mexico State, Catron Country, San Francisco River at Glenwood, 33°12'52"N, 109°28'01"W, 7.III.1976, reared, leg: Sta. H & M. Beard. ***Paratypes*.** 2 males, USA, New Mexico State, Catron Country, San Francisco River, 17.IX.1974, leg: M. Beard. 1 male, USA, New Mexico State, Catron Country, San Francisco River at Glenwood, 7.III.1976, reared, leg: Sta. H & M. Beard.

#### Diagnostic characters.

Thorax brown, with dark brown spots. Femora and tibiae of mid and hind legs dark brown at proximal and distal 1/5. The posterior margin of tergite IX cone-like; anal point slender, parallel-sided, apically rounded; superior volsella parallel-sided at base, slightly curved in the middle, widest at 1/3 distance from apex and apically swollen; inferior volsella thumb-like, with slight extension at base; gonostylus curved at 1/4 distance from base, parallel-sided towards apex, rounded apically.

#### Description.

**Male** (*n* = 4, unless stated).

Total length 4.24–5.05, 4.59 mm. Wing length 2.00–2.37, 2.12 mm. Total length/wing length 2.12–2.27, 2.17. Wing length/length of profemur 1.95–2.31, 2.20.

***Coloration*.** Thorax brown, with dark brown spots. Femora and tibiae of front legs dark brown except for yellowish brown at distal 3/4, tarsi I dark brown except for yellowish brown at basal, tarsi II–V dark brown; femora and tibiae of mid and hind legs both dark brown at both proximal and distal 1/5, yellowish brown in the middle, tarsi I yellowish brown except for dark yellowish brown at apex, tarsi II–V dark yellowish brown. Abdomen yellowish brown, hypopygium dark yellowish brown.

**Figure 13.**
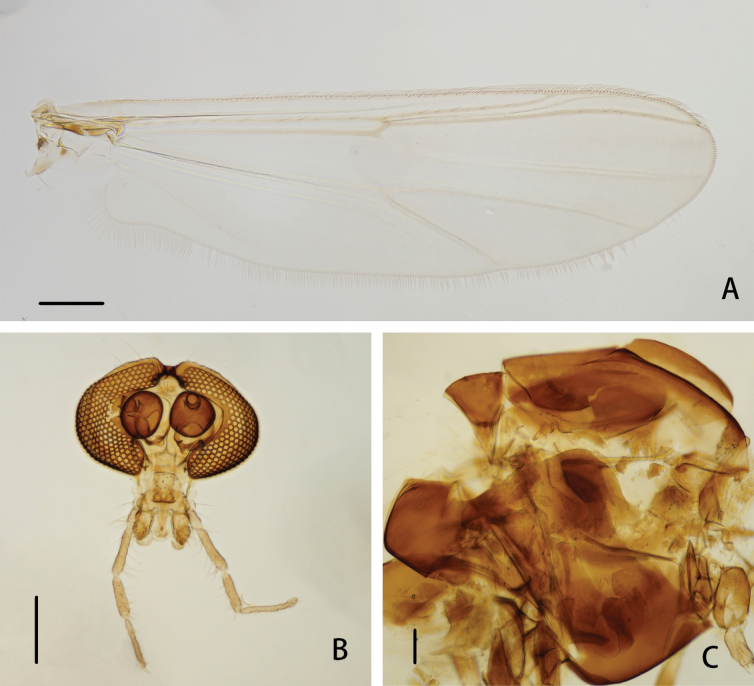
*Cryptochironomusparallelus* Liu, sp. nov., holotype male **A** wing **B** head **C** thorax. Scale bars: 200 μm.

***Head*** (Figs 13B, 15A). Antenna with 11 flagellomeres, ultimate flagellomere 697–802, 746 μm long. AR 1.99–2.37, 2.17. Frontal tubercles conical, 15–23, 19 (3) μm high, 10–14, 13 (3) μm width at base. Temporal setae 16–23, 18. Clypeus with 14–18, 16 setae. Tentorium 115–144, 135 μm long, 39–42, 41 μm wide. Palpomere lengths (in μm): 34–49, 40; 73–84, 79; 156–176, 169; 132–142, 137; 191–225, 211; Pm5/Pm3 1.14–1.35, 1.26.

***Thorax*** (Fig. 13C). Antepronotals bare; acrostichals 5–10, 8; dorsocentrals 8–11, 10; prealars 4. Scutellum with 14–16, 15 setae.

Wing (Figs 13A, 15D). VR 1.02–1.09, 1.06. R with 10–18, 14 setae, R_1_ with 6–10, 8 setae, R_4+5_ with 10–15, 12 setae. Brachiolum with three setae. Squama with 10–17, 14 (2) setae.

***Legs*.** Front tibia with three subapical setae, 114–121, 117.5 (2) μm, 129 (1) μm, 138 (1) μm. Combs of midtibia 41–57, 49 μm wide with 21–29, 24 μm long spur, and 42–61, 54 μm wide with 32–39, 35 μm long spur; combs of hind tibia 28–48, 36 μm wide with 28–36, 34 μm long spur, 80–92, 84 μm wide with 40–50, 44 μm long spur. Tarsus I of mid leg with three sensilla chaetica; tarsus I of hind leg with three sensilla chaetica. Lengths (in μm) and proportions of legs as in Table 5.

**Table 5.** Lengths (in μm) and proportions of legs of *Cryptochironomusparallelus* Liu, sp. nov., adult males (*n* = 4, unless stated).

	**fe**	**ti**	**ta_1_**	**ta_2_**	**ta_3_**
P_1_	871–1112, 945	675–770, 710	1091–1125, 1111 (3)	491–553, 520 (3)	383–431, 413(3)
P_2_	728–990, 831	753–872, 793	440–540, 477	224–306, 255	160–218, 191
P_3_	845–1083, 960	922–1124, 990	704–710, 707 (3)	338–382, 353 (3)	270–296, 281 (3)
	**ta_4_**	**ta_5_**	**LR**	**BV**	**SV**
P_1_	300–334, 316 (3)	153–167, 160 (3)	1.60–1.62, 1.61 (3)	1.85–1.99, 1.91 (3)	1.40–1.45, 1.42 (3)
P_2_	106–152, 124	89–120, 104	0.58–0.62, 0.60	3.01–3.42, 3.13	3.29–3.50, 3.40
P_3_	158–180, 168 (3)	107–109, 108 (3)	0.72–0.77, 0.75 (3)	2.81–2.83, 2.83 (3)	2.52–2.84, 2.64 (3)

**Figure 14.**
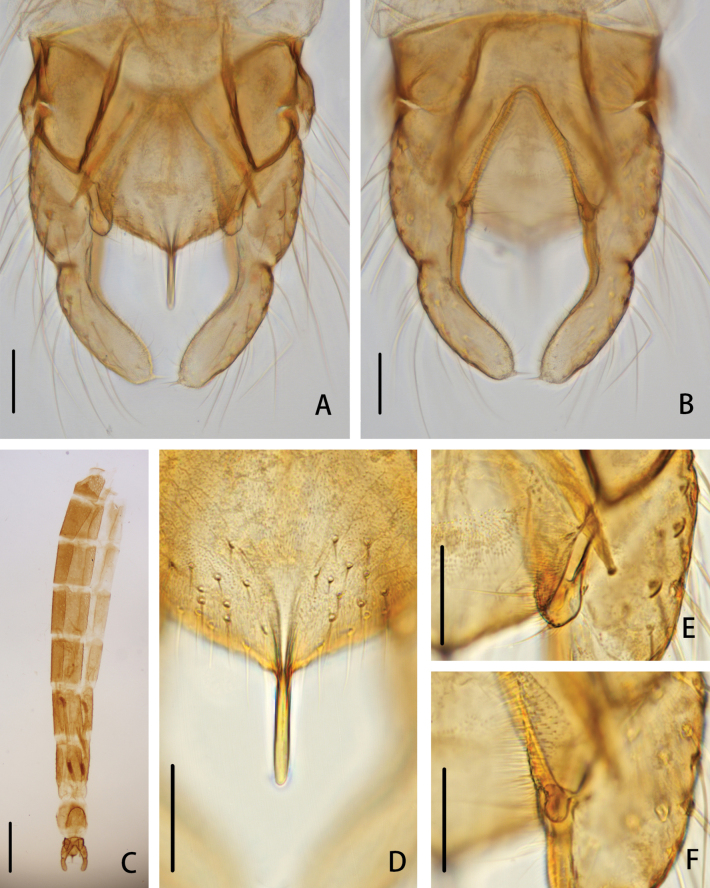
*Cryptochironomusparallelus* Liu, sp. nov., holotype male **A** hypopygium, dorsal view **B** hypopygium, ventral view **C** abdomen **D** anal point **E** superior volsella **F** inferior volsella. Scale bars: 50 μm (**A, B, D–F**); 500 μm (**C**).

**Figure 15.**
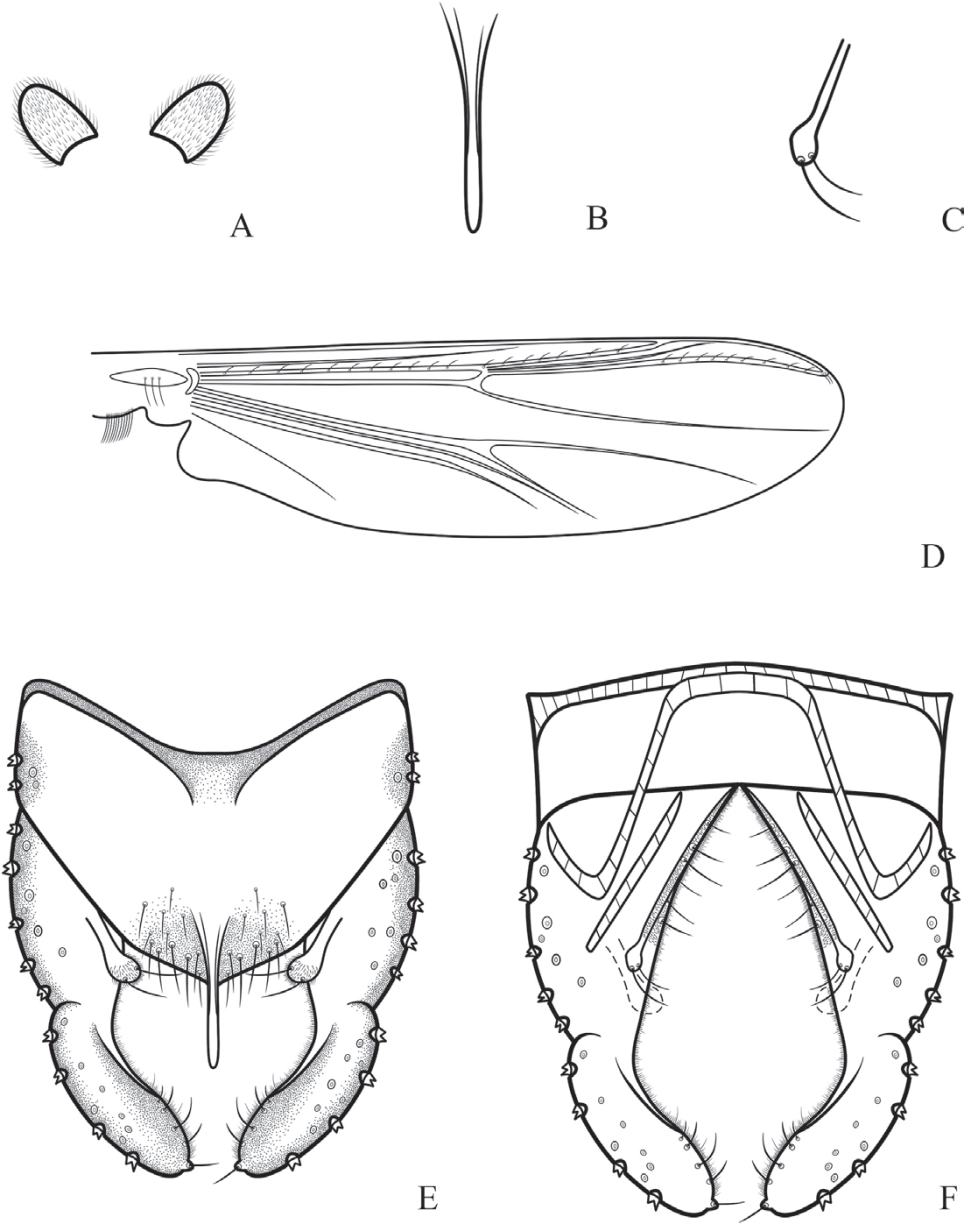
*Cryptochironomusparallelus* Liu, sp. nov., holotype male **A** frontal tubercles **B** anal point **C** inferior volsella **D** wing **E** hypopygium, dorsal view **F** hypopygium, ventral view.

***Hypopygium*** (Figs 14, 15B, C, E, F). The posterior margin of tergite IX is cone-like and bears ~ 22–36, 28 setae dorsally and ventrally near the base of the anal point. Laterosternite IX has 3–5, 4 setae. The anal point measures 54–65, 59 μm long and is slender with parallel sides, apically rounded. The anal tergite bands are V-shaped and show significant healing in the middle. The phallapodeme measures 103–120, 110 μm long, and the transverse sternapodeme is 56–89, 71 μm long. The superior volsella is 34–54, 47 μm long, 16–19, 17 μm wide, parallel-sided at the base, slightly curved in the middle, widest at the 1/3 distance from the apex, and apically swollen. It is covered with microtrichia and bears two strong setae at the apex. The inferior volsella is thumb-like, 15–20, 17 μm long, with a slight extension at the base and bears two setae at the apex. The gonocoxite measures 161–183, 176 μm long and bears seven strong setae along the inner margin. The gonostylus is 124–132, 127 μm long, curved at the 1/4 distance from the base, parallel-sided towards the apex, rounded apically, bears five short setae along the inner margin and one seta at the apex. HR 1.30–1.44, 1.38. HV 3.42–3.82, 3.60.

#### Etymology.

From the Latin *parallelus*, parallel-sided, referring to the character of anal point, adjective in the nominative singular.

#### Remarks.

*Cryptochironomusparallelus* Liu, sp. nov. resembles *Cryptochironomusdigitatus* Malloch, 1915 and *Cryptochironomusblarina* Townes, 1945 by having similar shapes of the posterior margin of tergite IX, inferior volsella and gonostylus. However, it can be distinguished from *C.digitatus* by the following combination of characters: small frontal tubercles, AR 1.99–2.37, Wing length 2.00–2.37, 2.12 mm, anal point 54–65, 59 μm long and is slender with parallel sides, apically rounded in *C.parallelus* Liu, sp. nov.; whereas large frontal tubercles, AR 3.14–3.23, superior volsella with a hook-shaped base, and anal point constricted at the 1/3 distance from the base in *C.digitatus*; whereas large frontal tubercles, AR 3.7, Wing length 3.5 mm, anal point 230 μm long and is gradually tapered and pointed apically in *C.blarina*.

### ﻿ Cryptochironomus taylorensis

Taxon classificationAnimaliaDipteraChironomidae

Liu sp. nov.

1C71C002-2BCD-5765-9BE9-1DEDF4BD20D1

https://zoobank.org/12993976-FF7D-4768-8C2A-8F9615128AC0

Figs 16 , 17 , 18

#### Type material.

***Holotype*.** one male, USA, New Mexico State, Colfax Country, Canadian River at Taylor Springs, 36°32'74"N, 104°49'22"W, 15.VII.1974, sweep net, leg: Sta. C. ***Paratypes*.** 2 males, New Mexico State, Colfax Country, Canadian River at Taylor Springs, 15.VII.1974, sweep net, leg: Sta. C.

#### Diagnostic characters.

Frontal tubercles absent; squama with 19 setae; anal point parallel-sided, slightly widening at apex; superior volsella crescent-like, with slender extension at the base, curved in the middle and apically swollen; inferior volsella triangular, widest at base, completely covered by superior volsella; gonostylus gradually tapered and pointed apically.

#### Description.

**Male** (*n* = 3, unless stated).

Total length 4.65–5.49, 5.01 mm. Wing length 2.31–2.59, 2.42 mm. Total length/wing length 1.98–2.12, 2.07. Wing length/length of profemur 2.00–2.79, 2.38.

#### *Coloration*.

Thorax light yellow-brown, with yellow-brown spots. Femora of front legs yellow-brown except for dark yellow-brown at both ends, tibia dark brown, tarsomeres lost; femora and tibiae of mid and hind legs yellow-brown, tarsus I–V yellow-brown to dark brown gradually. Abdomen yellow-brown, hypopygium dark brown.

**Figure 16.**
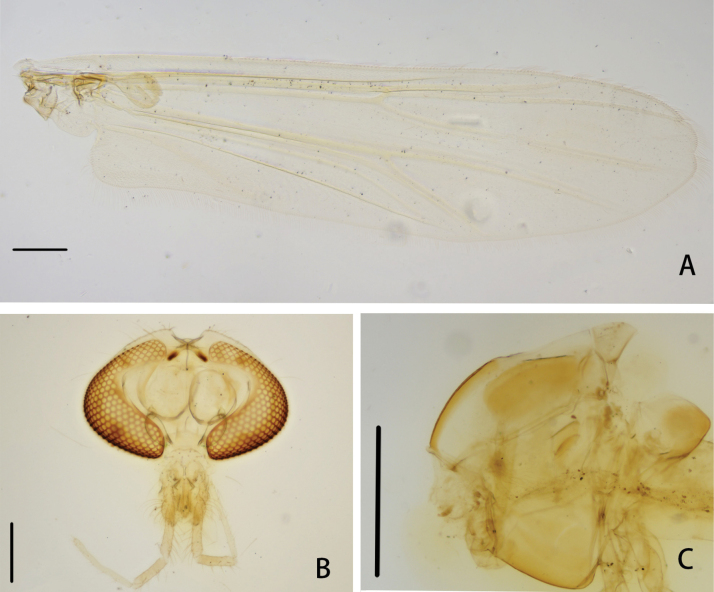
*Cryptochironomustaylorensis* Liu, sp. nov., holotype male **A** wing **B** head **C** thorax. Scale bars: 200 μm.

***Head*** (Fig. 16B). Antenna with 11 flagellomeres, ultimate flagellomere 861–1050, 980 μm long. AR 2.68–2.90, 2.82. Frontal tubercles absent. Temporal setae 16–18, 17. Clypeus with 15–17, 16 setae. Tentorium 131–176, 154 μm long, 50–61, 56 μm wide. Palpomere lengths of three specimens (in μm): 41–44, 43; 70–77, 74; 185–196, 191; 160–171, 166; 239–262, 251; Pm5/Pm3 1.29–1.34, 1.31.

***Thorax*** (Fig. 16C). Antepronotals bare; dorsocentrals 8–10, 9; acrostichals 4–5, 5; prealars 5. Scutellum with 10–12, 11 setae.

***Wing*** (Figs 16A, 18C). VR 1.05–1.11, 1.07. R with 15–18, 17 setae, R_1_ with 16–20, 18 setae, R_4+5_ with 21–22, 22 setae. Brachiolum with two setae. Squama with 19 (1) setae.

***Legs*.** Fore leg bearing three subapical setae, 142 (1) μm, 144 (1) μm, 146 (1) μm long. Combs of mid tibia 40–60, 52 μm wide with 24–34, 29 μm long spur, and 45–69, 60 μm wide with 43–56, 49 μm long spur; combs of hind tibia 51–56, 53 μm wide with 34–39, 37 μm long spur, 97–105, 100 μm wide with 51–56, 53 μm long spur. Tarsus I of mid leg with 3–5, 4 sensilla chaetica; tarsus I of hind leg with 3–5, 4 sensilla chaetica. Lengths (in μm) and proportions of legs as in Table 6.

**Table 6.** Lengths (in μm) and proportions of legs of *Cryptochironomustaylorensis* Liu, sp. nov., adult males (*n* = 3, unless stated).

	**fe**	**ti**	**ta_1_**	**ta_2_**	**ta_3_**
P_1_	830–1173, 1034	841–857, 848	1283 (1)	669 (1)	505 (1)
P_2_	815–1062, 950	899–952, 927	539–583, 556	242–267, 251	157–183, 167
P_3_	994–1142, 1050	1127–1285, 1197	807–817, 812 (2)	381–383, 382 (2)	302–310, 306 (2)
	**ta_4_**	**ta_5_**	**LR**	**BV**	**SV**
P_1_	418 (1)	203 (1)	1.53 (1)	1.65 (1)	1.30 (1)
P_2_	103–115, 109	88–108, 98	0.59–0.61, 0.60	3.79–4.03, 3.89	3.18–3.49, 3.37
P_3_	184–201, 193 (2)	114–129, 122 (2)	0.69–0.72, 0.70 (2)	2.95–2.97, 2.96 (2)	2.65–2.66, 2.66 (2)

**Figure 17.**
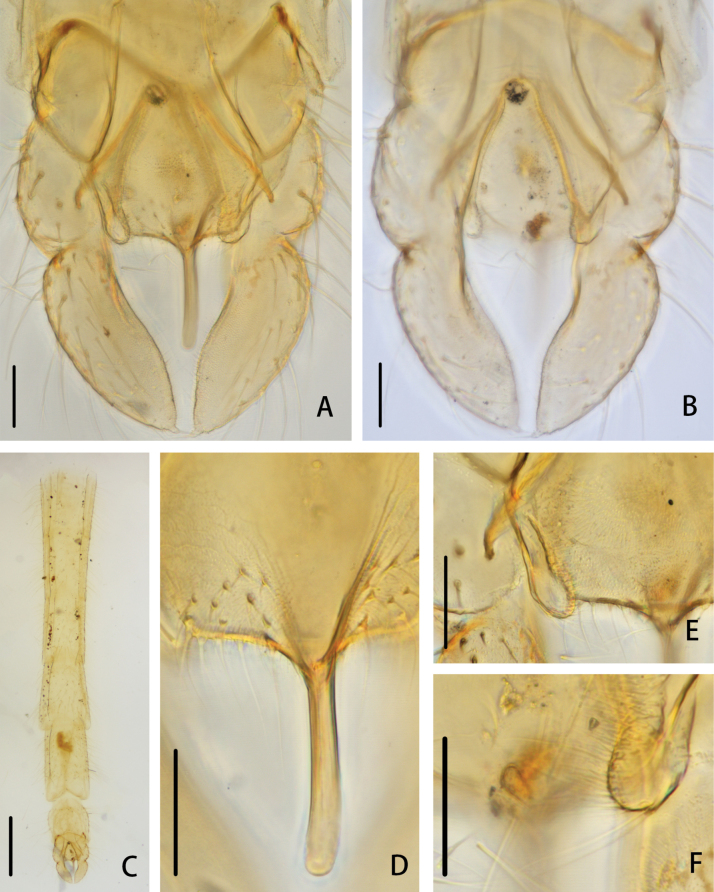
*Cryptochironomustaylorensis* Liu, sp. nov., holotype male **A** hypopygium, dorsal view **B** hypopygium, ventral view **C** abdomen **D** anal point **E** superior volsella **F** inferior volsella. Scale bars: 50 μm (**A, B, D–F**); 500 μm (**C**).

**Figure 18.**
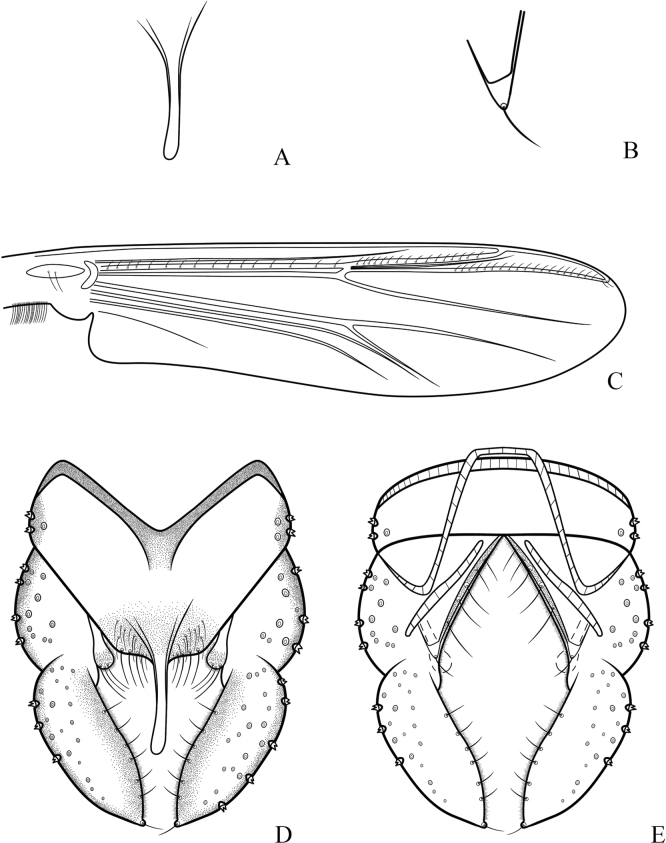
*Cryptochironomustaylorensis* Liu, sp. nov., holotype male **A** anal point **B** inferior volsella **C** wing **D** hypopygium, dorsal view **E** hypopygium, ventral view.

***Hypopygium*** (Figs 17, 18A, B, D, E). The posterior margin of tergite IX is shoulder-shaped and bears 30–32, 31 setae at the base of the anal point. Laterosternite IX has 3–4, 4 setae. The anal point measures 91–103, 96 μm long and is parallel-sided, widening slightly at the apex. The anal tergite bands are fused and V-shaped. The phallapodeme measures 131–147, 139 μm long, and the transverse sternapodeme is 65–92, 78 μm long. The superior volsella is 60–63, 62 μm long, 18–23, 21 μm wide, crescent-like, with a slender extension at the base, curved in the middle and apically swollen, and has 3 long setae at the apex. The inferior volsella is triangular, 19–24, 22 μm long, widest at the base, completely covered by the superior volsella, with one apically seta and without microtrichia. The gonocoxite measures 174–184, 179 μm long and bears 30–32, 31 strong setae along the inner margin. The gonostylus is 169–192, 183 μm long, gradually tapered and pointed apically, and bears one seta at the apex. HR 0.93–1.03, 0.98. HV 2.47–2.89, 2.74.

#### Etymology.

Name after the type locality, the Canadian River at Taylor Springs; noun in nominative case.

#### Remarks.

*Cryptochironomustaylorensis* Liu, sp. nov. bears resemblance to *C.curryi* Mason, 1986 due to its similar shapes of the anal point, superior volsella, and anal tergite bands. However, it can be distinguished from *C.curryi* by the following combination of characters: R with 15–18 setae, inferior volsella triangular, and gonostylus pointed apically in this new species; whereas R with 40–46 setae, inferior volsella finger-shaped, and gonostylus rounded apically in *C.curryi*.

### ﻿Key to Nearctic males of *Cryptochironomus* Kieffer (adapted from Silva et al. 2010)

**Table:** 

1	Anal point broad and flat	**2**
–	Anal point narrow	**3**
2	Mesal apical margin of gonostylus emarginate	***C.scimitarus* Townes, 1945**
–	Mesal apical margin of gonostylus straight or nearly straight	***C.sorex* Townes, 1945**
3	Mesal apical margin of gonostylus emarginate	***C.argus* Roback, 1957**
–	Mesal apical margin of gonostylus not emarginate	**4**
4	AR ~ 3.7, Wing length 3.1–3.4 mm	**5**
–	AR ~ 2.63, Wing length 2.43–2.65 mm	**6**
5	Anal point gradually tapered and pointed apically	***C.blarina* Townes, 1945**
–	Anal point widest at base	***C.beardi* Liu, sp. nov.**
6	Gonostylus widened towards apex, frontal tubercles present	**7**
–	Gonostylus not distinctly widened towards apex, frontal tubercles mostly absent	**9**
7	AR 4.35–5.12, LR_1_ 1.12–1.21, gonostylus strongly widened towards apex	***C.stylifera* Johannsen, 1908**
–	AR 1.99–4.03, LR_1_ 1.23–1.62, gonostylus slightly widened towards apex	**8**
8	Frontal tubercles large, AR 3.14–3.23, the posterior margin of tergite IX shoulder-shaped	***C.digitatus* Malloch, 1915**
–	Frontal tubercles small, AR 1.99–2.37, the posterior margin of tergite IX cone-like	***C.parallelus* Liu, sp. nov.**
9	LR_1_ 1.31–1.67, frontal tubercles absent	**10**
–	LR_1_ 1.45–2.02, when lower than 1.7 frontal tubercles present	**12**
10	Wing length 4.99–5.78 mm; LR_1_ 1.48–1.67	***C.eminentia* Mason, 1986**
–	Wing length 2.30–4.75 mm; LR_1_ 1.31–1.55	**11**
11	Wing length 3.01–4.75 mm, AR 2.64–3.40, LR_1_ 1.34–1.55	***C.ramus* Mason, 1986**
–	Wing length 2.31–2.59 mm, AR 2.68–2.90, LR_1_ 1.53	***C.taylorensis* Liu, sp. nov.**
12	Wing length 1.47–1.8 mm, gonostylus only ~ 2× as long as wide	**13**
–	Wing length 1.9–5.6 mm, gonostylus at least 2.5× as long as wide	**14**
13	Wing length 1.8 mm, AR 2.75, frontal tubercles present	***C.parafulvus* Beck & Beck, 1964**
–	Wing length 1.47–1.69 mm, AR 2.29–2.49, frontal tubercles absent	***C.ferringtoni* Liu, sp. nov.**
14	Gonostylus ~ 2.8–3.2× as long as wide, anal point slightly spatulate apically, frontal tubercles absent	***C.ponderosus* (Sublette, 1964)**
–	Gonostylus ~ 2.7× as long as wide, anal point tapering parallel-sided or slightly spatulate, frontal tubercles present or absent	**15**
15	Frontal tubercles absent, wing length 2.5–5.6 mm	**16**
–	Frontal tubercles present, wing length 5.1–5.3 mm	**17**
16	Gonostylus rounded apically, wing length 2.5–5.6 mm	***C.curryi* Mason, 1986**
–	Gonostylus tapering to the apex, wing length 2.0 mm	***C.absum* Liu, sp. nov.**
17	Wing length 5.1–5.3 mm, AR 2.5–2.6	***C.conus* Mason, 1986**
–	Wing length 1.8–3.2 mm; AR 2.5–3.4	**18**
18	LR_1_ 1.56–1.73, anal point slightly spatulate apically	***C.imitans* Sæther, 2009**
–	LR_1_ 1.60–2.02, anal point parallel-sided	**19**
19	Thorax with dark brown spots, inferior volsella not dentate at base	***C.fulvus* Johannsen, 1905**
–	Thorax without spots, inferior volsella dentate at base	***C.dentatus* Liu, sp. nov.**

## Supplementary Material

XML Treatment for Cryptochironomus absum

XML Treatment for Cryptochironomus beardi

XML Treatment for Cryptochironomus dentatus

XML Treatment for Cryptochironomus ferringtoni

XML Treatment for Cryptochironomus parallelus

XML Treatment for Cryptochironomus taylorensis

## ﻿Additional information

### Author ORCIDs

Wen-Bin Liu https://orcid.org/0000-0001-6916-0692

### Data availability

All of the data that support the findings of this study are available in the main text.
