# ﻿Corrigendum: Liu W-B, Wang C-Y, Tang Y-N, Wang Y, Pei W-X, Yan C-C (2024) Six new species of *Cryptochironomus* Kieffer (Diptera, Chironomidae) from the Nearctic region. ZooKeys 1200: 275–302. doi: 10.3897/zookeys.1200.119225

**DOI:** 10.3897/zookeys.1207.128152

**Published:** 2024-07-24

**Authors:** Wen-Bin Liu, Cheng-Yan Wang, Ya-Ning Tang, Ying Wang, Wen-Xuan Pei, Chun-Cai Yan

**Affiliations:** 1 Tianjin Key Laboratory of Conservation and Utilization of Animal Diversity, Tianjin Normal University, Tianjin, 300387, China Tianjin Normal University Tianjin China

We recently published six new species of *Cryptochironomus* Kieffer (Diptera, Chironomidae) from the Nearctic region. However, the authors made some errors in couplet 4, in that the values given in the second alternative of the couplet do not agree with in the least the values that appear further down the key (couplet 7, etc.), and parentheses were missing in authorities of three species. In this corrigendum, we revise the key to Nearctic males of *Cryptochironomus*. We thank Dr. Martin Spies (Zoologische Staatssammlung München, München) for kindly pointing out the error in contradictions between couplets.

## ﻿Key to Nearctic males of *Cryptochironomus* Kieffer

The key adapted from Sæther 2009.

**Table:** 

1	Anal point broad and flat	**2**
–	Anal point narrow	**3**
2	Mesal apical margin of gonostylus emarginate	***C.scimitarus* Townes, 1945**
–	Mesal apical margin of gonostylus straight or nearly straight	***C.sorex* Townes, 1945**
3	Mesal apical margin of gonostylus emarginate	***C.argus* Roback, 1957**
–	Mesal apical margin of gonostylus not emarginate	**4**
4	Gonostylus ~ 4× as long as wide, AR about 3.7, Wing length 3.1–3.4 mm	***C.blarina* Townes, 1945**
–	Gonostylus ~ 1.6–3.7× as long as wide; when AR higher than 3.6, Wing length higher than 3.4 mm or lower than 3.1 mm	**5**
5	Gonostylus widened towards apex; frontal tubercles present; AR 1.99–5.1; LR_1_ 1.12–1.73, 9–24 sensilla chaetica on P_2_	**6**
–	Gonostylus not distinctly widened towards apex; frontal tubercles mostly absent, when present AR 2.5–3.8, LR_1_1.45–2.02 and mostly 1–6 sensilla chaetica on P_2_; when AR higher than 3.5, LR_1_ higher than 1.59	**10**
6	AR 1.99–2.76; LR_1_ 1.60–1.7	**7**
–	AR 3.54–5.12; LR_1_ 1.12–1.39	**9**
7	Wing length 2.43–2.65; anal point widest at the base and slightly constricted at 1/3 distance from the base	***C.beardi* Liu, sp. nov.**
–	Wing length 2.05–2.37; anal point is parallel-sided with a rounded apex	**8**
8	AR 1.99–2.37; thorax brown with dark brown spots	***C.parallelus* Liu, sp. nov.**
–	AR 2.48–2.76; thorax yellowish brown without spots	***C.dentatus* Liu, sp. nov.**
9	AR 4.35–5.12, LR_1_ 1.12–1.21; gonostylus strongly widened towards apex	***C.stylifera* Johannsen, 1908**
–	AR 3.54–4.03, LR_1_ 1.23–1.39; gonostylus slightly widened towards apex	***C.digitatus* (Malloch, 1915)**
10	LR_1_ 1.31–1.67, frontal tubercles absent	**11**
–	LR_1_ 1.45–2.02, when lower than 1.7 frontal tubercles present	**13**
11	Wing length 4.99–5.78 mm; LR_1_ 1.48–1.67	***C.eminentia* Mason, 1986**
–	Wing length 1.47–4.75 mm; LR_1_ 1.31–1.55	**12**
12	Wing length 4.05 mm; gonostylus widest width near apex	***C.ramus* Mason, 1986**
–	Wing length 2.31–2.59 mm; gonostylus gradually tapered and pointed apically	***C.taylorensis* Liu, sp. nov.**
13	Wing length 1.47–1.8 mm, gonostylus only ~ 2× as long as wide	**14**
–	Wing length 1.9–5.6 mm, gonostylus at least 2.5× as long as wide	**5**
14	Wing length 1.8 mm, AR 2.75, frontal tubercles present	***C.parafulvus* Beck & Beck, 1964**
–	Wing length 1.47–1.69 mm, AR 2.29–2.49, frontal tubercles absent	***C.ferringtoni* Liu, sp. nov.**
15	Gonostylus ~ 2.8–3.2× as long as wide, anal point slightly spatulate apically, frontal tubercles absent	***C.ponderosus* (Sublette, 1964)**
–	Gonostylus ~ 2.7× as long as wide, anal point tapering parallel-sided or slightly spatulate, frontal tubercles present or absent	**16**
16	Frontal tubercles absent, wing length 2.0–5.6 mm	**17**
–	Frontal tubercles present, wing length 1.8–3.2 mm or 5.1–5.3 mm	**18**
17	Gonostylus rounded apically, wing length 2.5–5.6 mm	***C.curryi* Mason, 1986**
–	Gonostylus tapering to the apex, wing length 2.02 mm	***C.absum* Liu, sp. nov.**
18	Wing length 5.1–5.3 mm; AR 2.5–2.6	***C.conus* Mason, 1986**
–	Wing length 1.8–3.2 mm; AR 2.5–3.4	**19**
19	LR_1_ 1.56–1.73, mean 1.64; anal point slightly spatulate apically	***C.imitans* Sæther, 2009**
–	LR_1_ 1.60–2.02, mean 1.78, anal point parallel-sided	***C.fulvus* (Johannsen, 1905)**

## ﻿Additional information

### Author ORCIDs

Wen-Bin Liu https://orcid.org/0000-0001-6916-0692

### Data availability

All of the data that support the findings of this study are available in the main text.
